# Mechanical Agitation-Assisted Transmembrane Drug Delivery by Magnetically Powered Spiky Nanorobots

**DOI:** 10.34133/research.0768

**Published:** 2025-08-13

**Authors:** Xiaojia Liu, Zihan Xu, Yanan Che, Zichang Guo, Dongdong Jin, Qianqian Wang, Ning Liu, Xing Ma, Zhilu Yang

**Affiliations:** ^1^ Dongguan Key Laboratory of Smart Biomaterials and Regenerative Medicine, The Tenth Affiliated Hospital, Southern Medical University, Dongguan, Guangdong 523059, China.; ^2^ Sauvage Laboratory for Smart Materials, School of Integrated Circuits, Harbin Institute of Technology (Shenzhen), Shenzhen 518055, China.; ^3^ Department of Mechanical and Automation Engineering, The Chinese University of Hong Kong, Hong Kong, SAR 999077, China.; ^4^ Jiangsu Key Laboratory for Design and Manufacturing of Precision Medicine Equipment, School of Mechanical Engineering, Southeast University, Nanjing 211189, China.; ^5^ School of Mechanical Engineering, Southeast University (Jiulonghu Campus), Jiangning District, Nanjing 211189, China.; ^6^ School of Aerospace Engineering and Applied Mechanics, Tongji University, Shanghai 200092, China.

## Abstract

Breaking through cell membrane barriers is a crucial step for intracellular drug delivery in antitumor chemotherapy. Hereby, a magnetic nanorobot, capable of exerting mechanical agitation on cellular membrane to promote intracellular drug delivery, was developed. The main body of the nanorobots was composed of nano-scaled gold nanospikes that were deposited with Ni and Ti nanolayers for magnetic activation and biocompatibility, responsively. The nanorobots can be precisely navigated to target cancer cells under external magnetic field control. By virtue of the sharp nanospike structures, the magnetically powered rotation behavior of the nanorobots can impose mechanical agitation on the living cell membrane and thus improve the membrane permeability, leading to promoted transmembrane cargo delivery. Coarse-grained molecular dynamics simulation revealed that the mechanism of mechanical intervention regulated permeability of the bilayer lipid membrane, allowing for enhanced transmembrane diffusion of small cargo molecules. An in vitro study demonstrated that these nanorobots can markedly enhance the efficiency of drug entry into tumor cells, thus improving the effectiveness of tumor therapy under magnetic activation in vivo. This work paves a new way for overcoming cell membrane barriers for intracellular drug delivery by using a magnetic nanorobotic system, which is expected to promote further application of magnetically controlled nanorobot technology in the field of precision medicine.

## Introduction

Transmembrane drug delivery plays a pivotal step that fundamentally determines the therapeutic efficacy of chemotherapy in the treatment of various diseases. Whether combating cancer, neurodegenerative disorders, or infectious diseases, drug molecules must traverse the cell membrane barrier to reach their intracellular targets and exert their intended functions [1–3]. However, the cell membrane, as a natural selectively permeable barrier, not only segregates the intracellular and extracellular environments but also orchestrates molecular exchange through membrane-embedded machinery such as transmembrane proteins and ion channels [4,5]. This sophisticated defense system severely restricts the intracellular transport of exogenous agents. Currently, small-molecule drugs primarily rely on passive diffusion to cross the cell membrane. Yet, this process is inherently constrained by molecular size, hydrophobicity, and concentration gradients across the membrane, rendering it largely ineffective for macromolecular drugs and polar compounds [6,7]. Alternatively, nanocarriers can facilitate intracellular delivery via endocytosis by encapsulating therapeutic agents [8–10]. Nonetheless, this strategy faces significant challenges, including limited biocompatibility of the carriers, potential immunogenicity, and suboptimal release efficiency within the cell. More critically, in drug-resistant tumor cells, efflux transporters, such as ATP (adenosine triphosphate)-binding cassette proteins that can actively expel intracellular drugs, form a “drug efflux” barrier that substantially undermines the effectiveness of conventional delivery strategies [11]. The presence of this dual barrier underscores the urgent need to develop innovative transmembrane delivery mechanisms for overcoming current bottlenecks in drug delivery efficiency and enhancing clinical outcomes.

Micro/nanorobots refer to miniaturized structures and devices capable of autonomous motion and task execution in aqueous environments by converting various forms of energies into mechanical work [12,13]. In recent years, their potential applications in the biomedical field, particularly in drug delivery, have garnered considerable attention. By leveraging active transport and controlled-release mechanisms, micro/nanorobots offer promising strategies to overcome the limitations of conventional drug delivery systems, such as poor penetration across biological barriers and insufficient targeting capabilities [14–16]. For instance, Liu et al. [17] developed a microrobot-based system capable of actively delivering insulin, overcoming the harsh digestive environment and the absorptive barrier of the intestinal mucosa. Li et al. [18] constructed chemotactic nanorobots for targeted drug delivery in inflammation-related diseases. Jiang et al. [19] designed thermophoretic fluorescent nanorobots, offering a novel strategy for smart delivery in the photothermal treatment of superficial tumors. Additionally, Ma et al. [20] developed a tumor microenvironment-responsive aggregation-induced emission (AIE)-based nanocarrier system that effectively inhibits the overexpression of heat shock proteins, thereby enhancing the efficacy of mild-temperature photothermal therapy in tumor treatment.

Nevertheless, though significant achievements have been made by these studies, most of them paid few attention to the fundamental interactions between micro/nanorobots and tumor cell membranes. To date, several preliminary advances have been made in exploiting the mechanical motion of micro/nanorobots for transmembrane delivery. Smith et al. [21] utilized enhanced diffusive motion to increase collisions between cells and nanocarriers, thereby promoting cellular uptake. Chen et al. [22] developed dual-powered Janus nanorobots capable of modulating the hypoxic tumor microenvironment to improve photodynamic therapy efficiency, while simultaneously providing propulsion to facilitate deeper penetration of the nanorobots. Also, one-dimensional tubular nanorobots have been employed to construct biomimetic transmembrane nanochannels [23]. These systems, powered by enzyme-catalyzed reactions, demonstrated enhanced transmembrane drug delivery to drug-resistant tumor cells, although with limited control over transport dynamics. Sun et al. [24] engineered spiny, sunflower-pollen-derived magnetic microrobots capable of puncturing cancer cell membranes to deliver therapeutic agents directly. Despite these promising developments, the fundamental mechanisms underlying the mechanical interaction between micro/nanorobots and cell membranes remain poorly understood, which highlights an urgent need for detailed insights into the processes, by which how micro/nano-scaled mechanical forces perturb membrane structures and facilitate the permeation of small-molecule cargos.

**Fig. 1.**
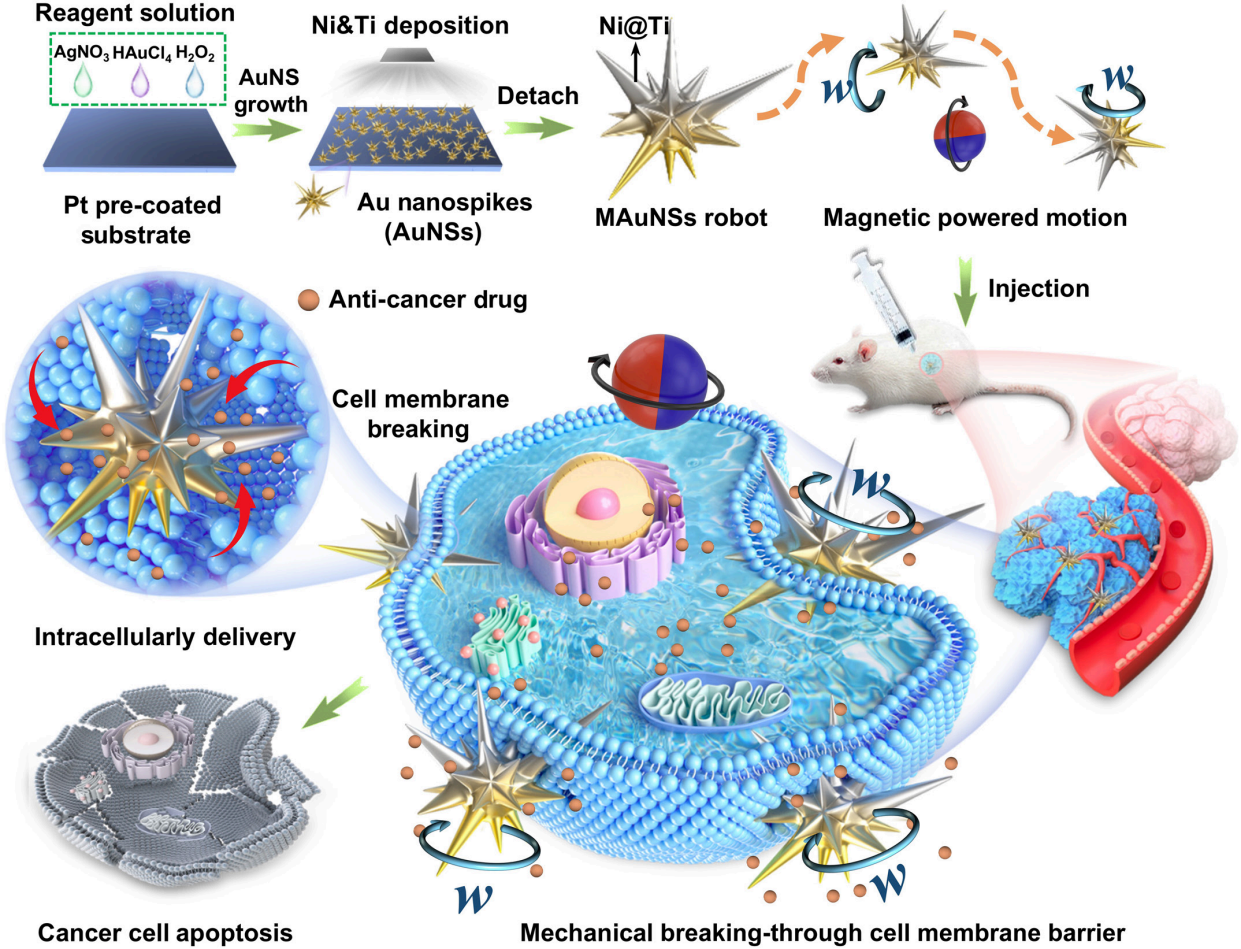
Scheme of the preparation of MAuNS robots, and their anti-cancer therapeutic applications by mechanically regulated cell membrane permeability for transmembrane drug delivery.

Herein, we have designed and developed gold nanospikes (AuNSs)-based magnetic nanorobots capable of conducting mechanical rupture of cell membrane for transmembrane drug delivery, as shown in Fig. 1. The AuNSs were rapidly synthesized in situ by dropwise addition of reaction solutions on a pre-coated Pt substrate according to our previous report [25]. Then, a Ni magnetic layer and a Ti protective layer are deposited on their surface to yield the magnetic nanorobots that can be activated to accomplish targeted motion and rotation on the cell membrane. Taking advantage of sharp edges of the nanospikes, the rotating nanorobots would impose mechanical agitation and even physical rupture effect on the cell membrane. Therefore, the permeability of the cell membrane was regulated, which allows therapeutic molecules to enter cells more efficiently, thus improving the effect of targeted cancer therapy in vivo. This work provides theoretical understanding of micro/nanorobotic system-enabled active and efficient drug transport across the cell membrane barrier, which is expected to bring insights into future biomedical applications of artificial micro/nano-machines.

## Results

### Synthesis and characterization of magnetic gold nanospikes robots

**Fig. 2.**
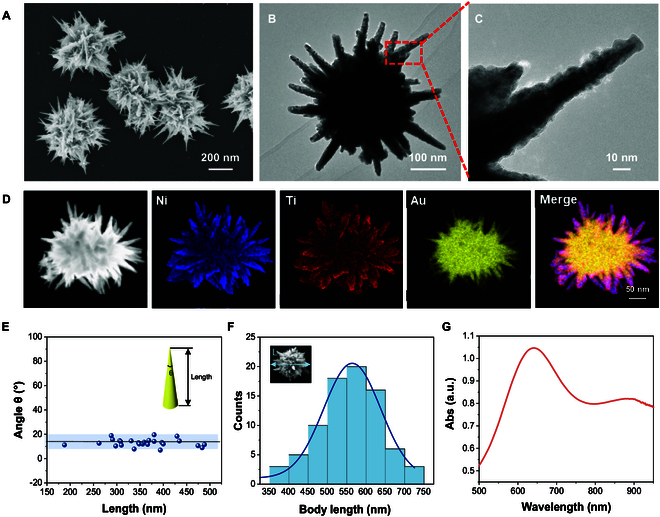
Characterization of MAuNS robots. (A) Scanning electron microscope (SEM) image of the MAuNSs. (B) Transmission electron microscope (TEM) image and (C) high-magnification image of the region marked by a red square of a single MAuNSs robot. (D) EDX mappings of a MAuNSs robot. (E) Plot showing the spine length and tip angle of the MAuNSs. (F) Statistics of the length of the MAuNSs. (G) UV–Vis spectrum of the MAuNSs suspended in deionized (DI) water.

Scanning electron microscope (SEM) images of the prepared magnetic gold nanospikes (MAuNSs) robots are shown in Fig. 2A, which have a relatively uniform size of about 500 nm. A “sea urchin-like” nanospikes structure with a size of about 500 nm was observed in the transmission electron microscope (TEM) image, where its sharp nanospike structures can be clearly identified as shown in Fig. S1. To endow the AuNSs with magnetic property, a layer of metallic Ni of about 40 nm was deposited on their surface by electron beam (e-beam) deposition, and subsequently, a layer of Ti of about 20 nm was further coated to protect the internal magnetic layer and obtain biocompatibility (Fig. S2). The MAuNSs robots were characterized by high-resolution SEM and TEM images (Fig. 2A to C), where the multi-spike structure was clearly visible. A single MAuNS particle was randomly selected for energy dispersive x-ray spectroscopy (EDX) elemental analysis, as shown in Fig. 2D and Fig. S3. The results showed that the MAuNSs particle had 3 elements, Au (yellow), Ni (blue), and Ti (red), in which the internal yellow AuNSs was the main structure, and the deposited Ni and Ti nanolayers covered the AuNSs and completely overlapped, which also implied the successful synthesis of the MAuNSs as designed. It is worth noting that the nanospikes were very sharp. The length and angle of a single nanospike were counted by ImageJ software’ Fig. 2E shows that the length of nanospikes is in the range of 150 to 500 nm, and the sharp angle is mainly concentrated in the range of 5° to 20°. Thus, the sharp nanospikes can act as miniaturized “scalpels” for mechanical treatment on tumor cell membrane. In addition, a region of 10 μm × 10 μm was further selected and the number of MAuNSs of different sizes, defined as the diameter of the MAuNSs, was counted as shown in Fig. 2F and Fig. S4. The length of the nanospikes located in 500 to 650 nm accounted for the largest proportion. Finally, as shown in Fig. 2G, in the ultraviolet–visible (UV-Vis) spectrum of the magnetic nanospikes, an absorption peak at 660 nm indicates the presence of gold nanostructures due to the surface plasmonic resonance effect. XPS spectra verified the presence of Ni and Ti elementary substance (Fig. S5). All these characterizations confirm the successful synthesis of MAuNS robots.

### Magnetic controlled motion behaviors of MAuNS robots

**Fig. 3.**
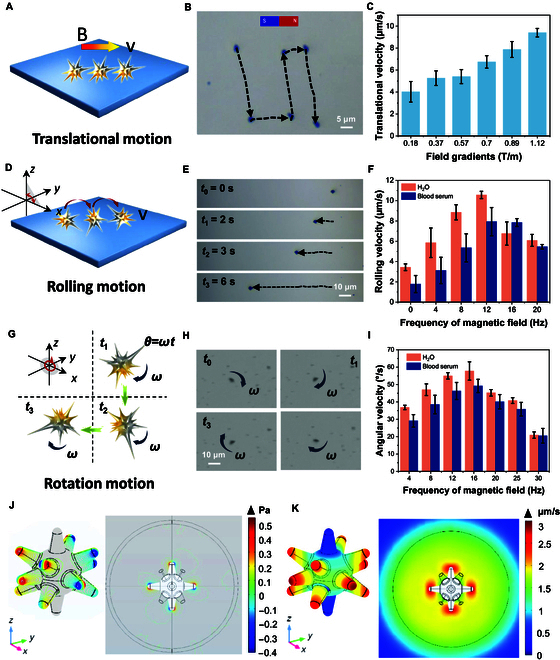
Motion control of MAuNSs robots by external magnetic field. (A) Schematic of the translational motion of MAuNSs robots driven by gradient field. (B) Optical microscope screenshot of the trajectory of a MAuNSs robot under a gradient magnetic field guidance. (C) Velocity of MAuNSs under different gradient magnetic field strengths (error bars indicate standard deviation, *n* = 50). (D) Schematic of the rolling motion of a MAuNSs robot. (E) Optical microscope screenshot of the trajectory of a rolling nanorobot at different time intervals. (F) Velocity of MAuNSs’ rolling motion under different magnetic field frequencies (error bars indicate standard deviation, *n* = 50). (G) Schematic of the rotation of a MAuNS robot with an applied XOY magnetic field. (H) Optical microscope screenshot of rotation nanorobots at different time intervals. (I) Rotational angular velocity of magnetically controlled robot motion at different magnetic field frequencies (4 to 30 Hz) (error bars indicate standard deviation, *n* = 50). (J) Simulation results of the velocity and direction of the fluid field around the nanospike under translational motion and (K) rolling motion.

The magnetically powered mechanical motion behavior of the MAuNS robots was studied by using a self-developed 3-dimensional (3D) magnetic field control system (Fig. S6). As shown in Fig. 3A, under a gradient magnetic field, the MAuNSs robots perform a translational movement along the direction of the gradient due to magnetic attraction force. The microscope observation clearly shows that the motion of the MAuNSs robots can respond quickly to the change of the strength and direction of the magnetic field without any obvious delay, which demonstrates the sensitivity of the nanorobots to the external magnetic field, and thus guaranteeing for the precise control of the nanorobots in complex tasks (Fig. 3B and Movie S1). The velocity of the magnetic nanobots is increasing proportionally with the increase of the strength of applied external magnetic field (Fig. 3C). The fastest average velocity reached as high as 9.40 ± 0.38 μm/s, when the magnetic field gradient was about 1.12 T/m. In addition, MAuNSs robots move at different speeds in different fluid environments (fetal bovine serum [FBS], polyethylene glycol, and deionized [DI] water) because of fluid viscosity, interfacial interactions, and the strength of the ions in the fluid. Nevertheless, in general, the nanorobots are still well controlled in various fluid environments (Fig. S7).

Then, we used a rotating magnetic field parallel to the *X*–*Z* plane to evaluate the rolling behavior of the nanorobots (Fig. 3D). By regulating the rotational direction and rotational frequency of the external magnetic field, the rotational motion behavior of the nanorobots can be controlled in real time. The video screenshot showing the motion behavior of the nanospike nanorobots in the rotating magnetic field with a frequency of 4 Hz was presented, and the black dashed line indicates the motion trajectory of the robot within 6 s (Fig. 3E and Movie S2). Meanwhile, its translational velocity by rolling motion was tracked and analyzed using a self-developed code as shown in Fig. 3F. The average velocity of the MAuNSs robots generally shows an increasing trend when the frequency increases. The trend is like the case in serum, but serum has higher viscosity and resistance than DI water, so the speed is lower than that in water. However, when the magnetic field frequency increases to 12 Hz, the velocity reaches its maximum value. Then, the velocity decreases slightly when the frequency continues to increase from 12 to 20 Hz. It can be attributed to the “step out” effect when the mechanical rolling cannot keep up with the rotational frequency of the applied magnetic field, which is a common phenomenon for rolling behavior of micro/nanorobots. [26]

Then, we evaluated the self-rotation behavior of the nanorobots driven by a rotating magnetic field parallel to the *X*–*Y* plane, schematically shown in Fig. 3G. When the rotating magnetic field is applied, the MAuNSs robots can effectively overcome the viscous resistance of the surrounding medium and begins to rotate. The screenshots of the video are shown in Fig. 3H. The angular velocity reaches a maximum value of about 59°/s when the magnetic field frequency is 16 Hz and continues to decrease due to the step out effect as well (Fig. 3I and Movie S3). A similar trend was shown in the blood serum. Meanwhile, we employed finite element modeling to analyze mechanical perturbations generated by a rotating spiky nanorobot in an unbounded fluid domain. At a rotational frequency of 16 Hz, the nanostructure exhibited distinct hydrodynamic characteristics, with the spike tip demonstrating exceptional mechanical performance. The maximum localized pressure reached approximately 0.55 Pa at the tip region, approximately 450% greater than baseline values observed in other structural components (Fig. 3J). Simultaneously, tip-associated vortex velocities peaked at 7.7 μm/s, representing a 5.1-fold enhancement compared to the inner smooth region (Fig. 3K). This pronounced mechanical amplification at the spiked interface suggests an efficient mechanism of targeted cellular membrane perturbation, highlighting the critical role of surface topography in nanorobotic design.

### Single-cell targeting and mechanical motion promoted intracellular drug delivery by MAuNS robots

**Fig. 4.**
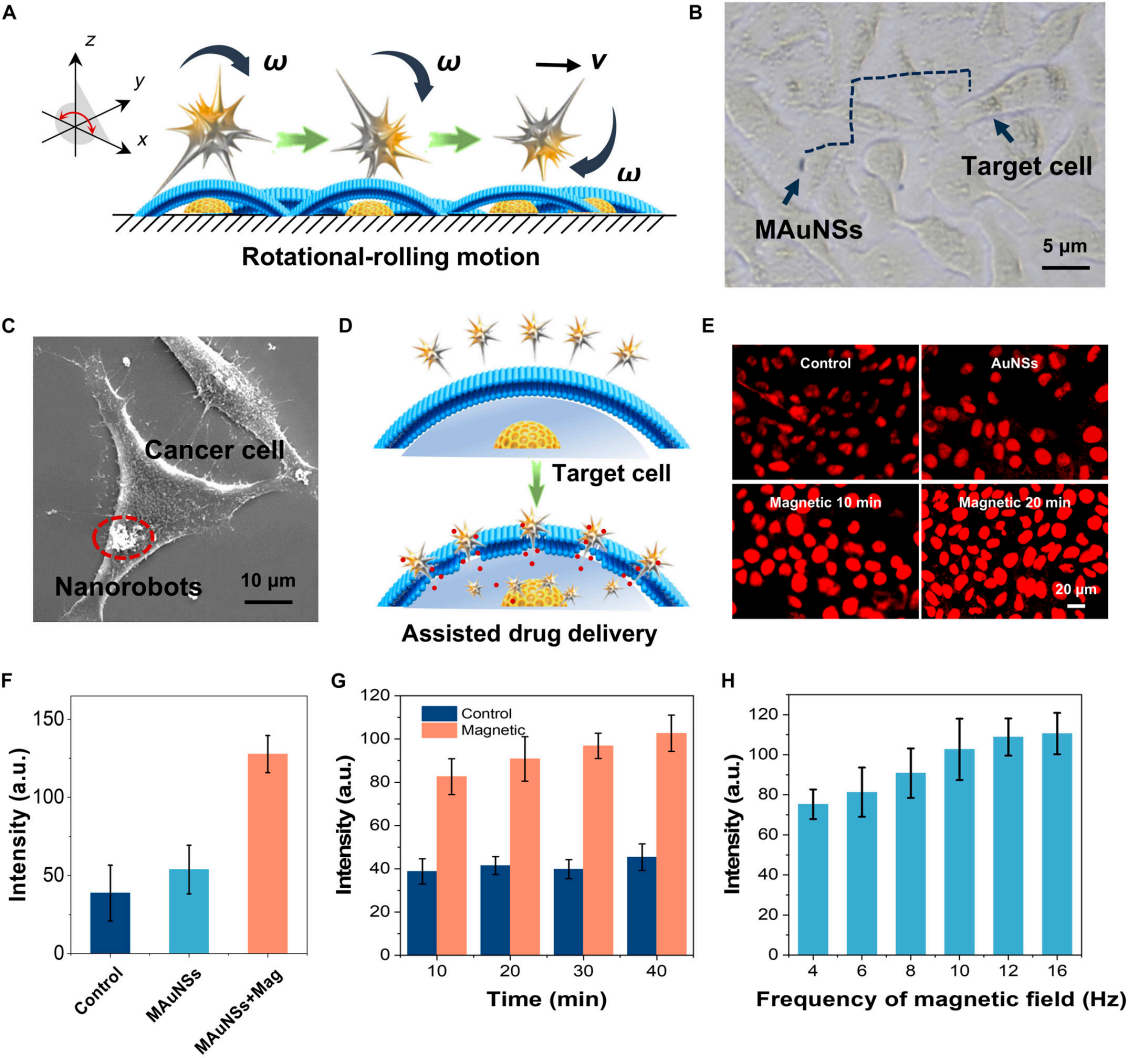
Magnetic field driven cell targeting and mechanical agitation enabled transmembrane drug delivery by MAuNSs robots. (A) Schematic diagram of a MAuNSs robot rolling forward driven by rotational magnetic field. (B) Optical microscopy screenshot capturing a MAuNSs robots targeting tumor cell (HepG-2) membranes in a cellular environment. (C) SEM image of cells (MAuNSs: 1 mg/ml; magnetic field exposure time: 30 min; red dotted circle indicates the presence of the MAuNSs). (D) Schematic diagram showing that mechanical agitation promoted transmembrane drug delivery. (E) Fluorescence images and (F) intensity analysis of drug molecules entering cells under different conditions. (G) Fluorescence intensity of drug molecules entering the cell at different times (10 to 40 s) and (H) different rotation frequencies (4 to 16 Hz) (error bars indicate standard deviation, *n* = 10).

To study the mechanical breaking through of the cell membrane by MAuNSs robots, we carried out an in vitro experiment to verify the mechanical motion-assisted transmembrane drug delivery on precisely targeted cells. First, a rotational rolling magnetic field was applied to drive a single nanorobot moving forward by the rolling effect (Fig. 4A). The MAuNSs robot was magnetically powered to move toward the targeted cell, and the movement trajectory was captured as shown in Fig. 4B and Movie S4. At the same time, we performed SEM observation on the targeted cells, where the MAuNS robots were attached onto the surface of the tumor’s cells (Fig. 4C). The presence of nanorobots on the targeted cells was also identified by confocal laser scanning microscope (CLSM) imaging (Fig. S8). The results proved that the nanorobots can effectively target cancer cells and bind onto the tumor cells’ membrane by magnetic field control.

COMSOL simulation was used to analyze the magnetic flux density generated by the MAuNSs robots after applying a rotating magnetic field in the *X*–*Y* axis. As shown in Fig. S9, the magnetic flux density on the surface of the MAuNSs is slightly shifted with any time change, while the maximum magnetic field strength is generated at the position of the spike tip. In addition, the propulsive force generated from the magnetic driving is balanced by the resistance applied to the MAuNSs. The pressure induced by the cell membrane is distributed over the half-side-rotating nanospike, and the tip location is subjected to high stresses from forward motion. This rotational motion behavior-induced force effect would endow the MAuNSs robots with mechanically killing capability toward cancer cells.

We anticipated that the magnetic field provides a driving force for the MAuNSs’ sharp spikes, which can effectively disrupt the cell membrane and create instanton “pores” or damage on the integration of the bilayer lipid cell membrane. Such mechanical interference would change the permeability of the cell membrane and thus makes it easier for small molecules to pass through the cell membrane for intracellular delivery, as shown in Fig. 4D. Fluorescence microscopy was used to observe the fluorescence intensity of antitumor drugs entering the tumor’s cells (HepG-2) (Fig. 4E). Meanwhile, the fluorescence intensities inside cells were quantitatively analyzed. With the mechanical agitation of the MAuNSs driven by the magnetic field (experimental group), the fluorescence intensity of cargo molecules, doxorubicin (DOX) used here, was significantly higher than that of the group without magnetic field (Fig. 4F). With the extension of the applying time of the magnetic field, the intensity of the drug entering the tumors cells is also significantly increased (Fig. 4G). Similarly, with the increase of the frequency of the applied rotating magnetic field, the intensity is also observed to increase (Fig. 4H). Besides, we tested it with different tumor cells (HeLa and CT26), and the results, as shown in Fig. S10, have a similar increasing trend of fluorescence intensity as compared to that of HepG-2 cells, suggesting the applicability of the mechanical stimulation-induced transmembrane delivery. On the one hand, the tangential force generated by the rotating and rolling of the MAuNSs makes the surrounding fluid circulation more conducive to the exchange of substances between the intracellular and extracellular environments. This promotes the diffusion of drug molecules and increases the efficiency of drug uptake by the cells. On the other hand, mechanical rupture of the membrane may change the microenvironment around the cell membrane. During the membrane-breaking process, charge distributions and lipid molecular arrangements on the surface of the cell membrane may be altered, which will attenuate the repulsive effect of the cell membrane on small-molecule drugs. Also, the MAuNSs robots will act as a “magnetic scalpel” to produce mechanical cutting force to damage the structure of cancer cells as shown in Fig. S11. With the increase of rotational frequency, the cell survival rate generally showed a decreasing trend. Calcein-AM and propidium iodide (PI) solutions were used to stain both live and dead cells, and a CLSM was used to observe both live (green) and dead (red) cells (Fig. S12). The cells in the phosphate-buffered saline (PBS) solution as a control group showed obvious green fluorescence, whereas almost all cells were observed to show red fluorescence after the addition of a rotating magnetic field, thus demonstrating the efficient mechano-killing therapeutic effect of the MAuNSs robots. By comparing the existing technologies, mechanical agitation-assisted transmembrane drug delivery provides a “non-chemically dependent, high-precision, low-risk” solution for biomedicine, which has great potential for application in the field of drug resistance and precision medicine. It has great potential for application in the field of drug resistance and precision medicine.

### Coarse-grained molecular dynamics simulations on mechanical agitation-assisted transmembrane cargo transport

**Fig. 5.**
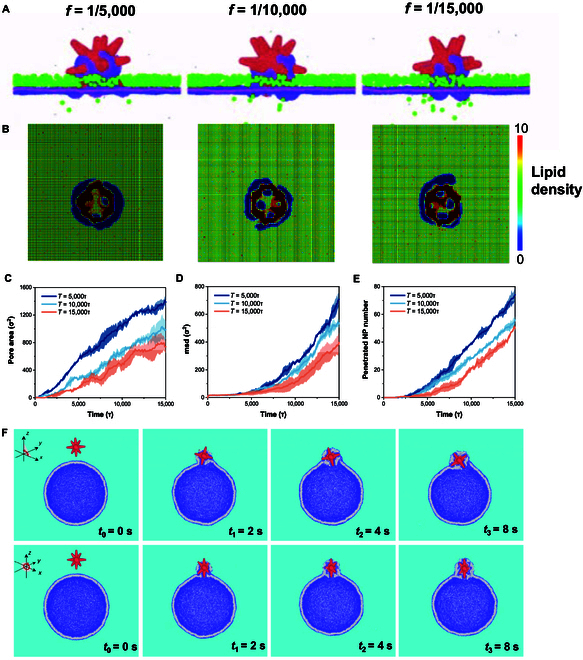
Coarse-grained molecular dynamics simulations. (A) Simulation of diffusion of small molecules at different rotational frequencies and (B) the corresponding lipid number density on the cell membrane. (C) Pore area, (D) mean square displacement, and (E) penetrated NP number produced by small molecules crossing the cell membrane at different rotational frequencies. (F) Screenshots of the dynamic behavior of MAuNS robots crossing the cell membrane at different times (*t*_0_ = 0 s, *t*_1_ = 2 s, *t*_2_ = 4 s, and *t*_3_ = 8 s).

Coarse-grained molecular dynamics simulations were performed to investigate the mechanism of rotation motion-induced change of the membrane permeability and thus promoted transmembrane drug delivery efficiency by the MAuNSs robots (in red color). Simulated nanoparticles (NPs) sized 2 nm was used as model drugs in the molecular dynamics’ simulation. As shown in Fig. 5A and Fig. S13, some of the NPs successfully penetrate the membrane due to free diffusion. However, for different rotating frequency conditions, the transmembrane transport efficiency of the NPs is different. At first glance, for *f* = 1/5,000τ^−1^, the NPs that have penetrated through the membrane are much more than that for *f* = 1/15,000τ^−1^. Figure 5B shows the lipid number density on the cell membrane, representing membrane conformation under the rotation of MAuNSs. Due to the spiny structure, the rotation of MAuNSs apparently brings “pores” to the modeled cell membrane. Figure 5C shows the variation of the number of NPs crossing the membrane. At the early stage of simulations, the number of transmembrane NPs experiences almost no changes due to the pore-free structure of the membrane as shown in Fig. 5E. Simultaneously, the mean square displacement along the transmembrane direction experiences marginal changes as shown in Fig. 5D. Later, the transmembrane NPs increase intensively as shown in Fig. 5C. At the same time, the mean square displacement along the transmembrane direction also increases sharply as shown in Fig. 5D. The above increase of transmembrane NP number and the transmembrane mean squared displacement can be attributed to the increase of pore size on the membrane as shown in Fig. 5E.

Figure 5F shows the dynamic rupture process of nanospike interacting with the cell membrane. It is assumed that the cell membrane is uniformly intact before interacting. When the MAuNSs attaches to the cell membrane, the receptor on the surface of the cell membrane moves toward the nanospike and eventually binds to the ligand on the surface of the MAuNSs. The ligand–receptor interaction causes localized lipid molecules in the membrane to follow the rotation of the nanospike, thereby generating shear forces within the lipid bilayer (Movies S5 and S6). Our results suggest that rotating the MAuNS leads to a higher likelihood of cell membrane rupture than a non-rotated one (Fig. S14 and Movie S7).

### Synergetic antitumor treatment by MAuNSs robots in vivo

**Fig. 6.**
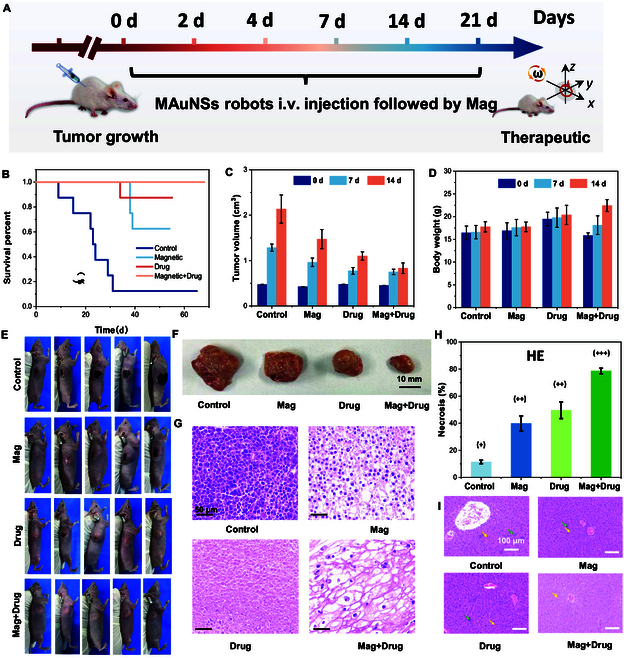
In vivo cancer therapy by synergetic antitumor effect of the MAuNSs robots. (A) Schematic illustration of the antitumor animal experiments. HepG-2 cells and MAuNSs were subcutaneously injected in turn into normal mice followed by magnetotherapy. (B) Survival of mice at different days. (C)Tumor volume (tumor volume divided by initial volume) in various mice groups. (D) Change in weight of mice in different groups over time. (E) Representative photographs of mice. (F) Morphologies of tumors at the original cell injection site. (G) H&E-stained images of tumors at the original cell injection site. (H) Necrosis in various mice groups. (I) H&E-stained images of liver tissue (error bars indicate standard deviation, *n* = 10).

Encouraged by previous findings, we anticipated that the rotational behavior induced mechanical agitation on cell membrane would not only cause mechanical damage to the cancer cells, but also promote intracellular delivery of anti-cancer drugs, leading to synergetic cancer therapy efficacy. Thus, in vivo antitumor efficacy of MAuNS robots was further investigated with HepG-2 xenograft liver tumor-bearing BALB/c mice (female, 6 weeks). The mice were all injected with MAuNS robots and divided into 4 groups (*n* = 10) with different treatment conditions: (a) Control (without any treatment), (b) Magnetic (apply magnetic field only), (c) Drug (DOX) (apply anti-cancer drug DOX only), (d) Magnetic+Drug (apply both magnetic field and drug), and the mice were administrated on days 0, 2, 4, 7, 14, and 21, respectively (Fig. 6A). After injection, a rotational magnetic field was applied to the tumor area for 20 min to propel the MAuNSs for mechanical killing of tumor cells. The tumor sizes and body weights were recorded every 7 days for 21 days, after which the tumors and major organs were collected for sectioning and hematoxylin and eosin (H&E) staining. A 50-day independent experiment of survival recording was performed to observe the survival rate (Fig. 6B). The survival rates of mice of the Magnetic+Drug group were improved to 100%, compared with 90% and 60% in the Drug and Magnetic groups, respectively, demonstrating the synergistic tumor-killing effect by chemotherapy and mechanotherapy effects. In contrast, the survival rate of the Control group was only 10% due to the systemic toxic effects and adverse reactions. As shown in Fig. 6C to F, the combination of DOX with magnetic field displayed the best antitumor efficacy and the tumor volume was suppressed considerably by 61.1%, when compared with that of the Control group. Compared to the Drug group (48.5%) and Magnetic group (31.1%), the Magnetic+Drug group exhibited a more significant tumor suppression effect, with a statistically significant difference. This could be attributed to MAuNS robots causing mechanical damage to the tumor tissue under magnetic field, facilitating the entry of the chemotherapeutic drug into tumor cells, resulting in a synergistic effect that significantly enhanced tumor treatment efficacy. Furthermore, compared to the other treatment groups, mice in the Magnetic+Drug group showed a noticeable increase in body weight, possibly due to the substantial tumor suppression efficacy.

To further analyze the apoptotic status of tumors in different treatment groups, H&E staining of collected tumors showed that the extracellular matrix (ECM) became loose with more extracellular space in the Magnetic and Magnetic+Drug groups, as compared to other groups without magnetic field exposure (Fig. 6G), indicating that spiky nanorobots with magnetic field can disrupt components of the ECM. In addition, compared to the Drug (49.5%) and Magnetic (39.8%) groups, the Magnetic+Drug group exhibited more necrotic tumor and stromal cells with karyorrhexis, with an apoptosis rate of 78.6% (Fig. 6H). Moreover, H&E staining of main organs including the heart, liver, spleen, lung, and kidney was then conducted. As shown in Fig. 6I, except for the Magnetic+Drug group, other groups all exhibited metastatic liver cancer cells in the liver due to insufficient tumor suppression. Additionally, no obvious morphological changes and inflammatory infiltrates were observed in other organs (Fig. S15), and blood biochemistry tests show that liver and kidney function indicators (e.g., alanine aminotransferase, aspartate aminotransferase, and creatinine) are within normal reference ranges, indicating the long-term biosafety of MAuNS robots (Fig. S16).

## Conclusion

Here, we propose a strategy to mechanically disrupt tumor cell membranes and assist drug delivery using magnetic field-driven MAuNS robots. Through experimental observation and data analysis, it is found that the AuNSs-modified robots can precisely target cancer cell membranes and achieve killing efficacy under magnetic field manipulation. By virtue of its unique structure and physical properties, MAuNSs could effectively destroy the integrity of the membranes to regulate membrane permeability, which greatly facilitates drug molecules to enter the cells across the cell membrane barrier. The fundamental mechanism of such mechanical agitation-assisted transmembrane cargo transport effect was theoretically analyzed through coarse-grained molecular dynamics simulation. Based on such achievements, the synergetic antitumor capability of the MAuNSs robots by mechanical and chemotherapy was verified in vivo. In the future, we will further optimize the preparation process of nanospike robots and explore the biological mechanism of micro/nanorobots to precisely target tumor cells. This work not only gives insights into the mechanical interaction between artificial micro/nanorobots and living cells, but also is expected to promote the biomedical use of magnetic micro/nanorobotic systems in cancer treatment.

## Methods

### Materials and characterization

In the process of fabrication of MAuNSs robots, silver nitrate solution (AgNO_3_), chloroauric acid (HAuCl_4_, 48% to 50% Au basis), hydrogen peroxide (H_2_O_2_), and ethanol (EtOH, 99.7%) were commercially purchased and used as received. In the cytotoxicity testing, Dulbecco’s modified Eagle medium (DMEM), trypsin, antibiotic, FBS, and PBS (pH 7.2) were commercially purchased from Thermo Fisher Scientific; Cell Counting Kit-8 (CCK-8; Zeta-life), Calcein-AM/PI double dye kit (Beyotime Biotechnology Co., Ltd.), and glutaraldehyde (50%, Aladdin) were commercially purchased and used as received.

X-ray diffraction data and Fourier-transformed infrared spectra were obtained using an x-ray diffractometer (D/Max-2500/PC) and a Fourier-transformed infrared spectrometer (Nicolet380, Thermo), respectively. The size distribution was measured by dynamic light scattering with Zetasizer Nano ZEP. SEM images and fluorescence images were captured by Phenom Microscopy (ProX) and a Leica inverted optical microscope (DMi8), respectively. TEM images and corresponding EDX spectroscopy were obtained by Talos F200x. Magnetic property was tested by a Vibrating Sample Magnetometer (Lake Shore 8600).

### Preparation of MAuNS robots

Typically, a sonicator (120 s) and a plasma cleaner (180 s) were used to clean the silicon wafer. Then, a layer of platinum was then deposited on the cleaned wafer by ion sputtering with a sputtering time of 40 s. Also, AgNO_3_ solution (20 μl, 10 mM) and HAuCl_4_ solution (60 μl, 50 mM) were added dropwise. After waiting for 20 s, the silicon wafer was rinsed with DI water. Ni and Ti nanolayers were deposited on the surface by electron beam vapor deposition, and the MAuNS robots were obtained for further use.

### Design of magnetic field control device

In this experiment, a 3D Helmholtz coil was designed and fabricated with a high-power DC power supply and a DC servo driver as the current drive source, an embedded virtual instrument platform as the controller, and a cartridge microscope and a charge-coupled device as the observation and video recording device. The design parameters of the 3D Helmholtz coil are shown in the Supplementary Materials.

### Magnetically controlled behavior of MAuNS robots and optical video recording

The motion control experiments of MAuNSs robots were carried out in a square quartz tank with a size of 22 mm × 22 mm × 5 mm. DI water (1 ml) was added to the quartz tank, and then 1 μl of MAuNSs robots dispersant with a concentration of 1 mg/ml was added to the DI water dropwise, and the quartz tank was placed in the center of a 3D Helmholtz coil to carry out the experiments. Also, the preparation of nanorobots was observed using a Leica inverted optical microscope with a 20× air objective.

### Model of lipid bilayers

A solvent-free coarse-grained molecular dynamics (CGMD) model for lipids developed by Cooke and Deserno [27], widely implemented to study interactions between membrane and NPs, is implemented here for our current CGMD simulations to construct a rectangular section of a lipid bilayer [28–36]. The lipid bilayer of size 200σ × 200σ is constructed. Each coarse-grained lipid molecule is composed of 3 connected beads. The blue bead represents the hydrophilic lipid head while the 2 white beads compose the hydrophobic lipid tail. The size of each bead type is determined by a Weeks–Chandler–Anderson potential:

$$U_{\mathrm{rep}}=\left\{\begin{array}{c} 4\varepsilon_{r}\left[{\left(\frac{b_{\text{head}\left(\text{tail}\right),\text{head}\left(\text{tail}\right)}}{r}\right)}^{12}-{\left(\frac{b_{\text{head}\left(\text{tail}\right),\text{head}\left(\text{tail}\right)}}{r}\right)}^{6}\;\right],r\leq r_{c}\\ 0,r>r_{c} \end{array}\right.$$ (1)

where $b_{\text{head},\text{head}}\;=\;b_{\text{head},\text{tail}}\;=\;0.95\;\sigma$, $\;b_{\text{tail},\text{tail}}\;=\;1.0\;\sigma$, and $\;\varepsilon_{r}\;=\;0.01\;\varepsilon$. An attractive interaction exists between tail beads to simulate the hydrophobic properties of the tail groups:

Ucos=−ε,r<rc−εcos2πr−rc2wc,rc≤r≤rc+wc0,r>rc+wc (2)

where $r_{c}=2^{1/6}\sigma$, and $w_{c}=1.7\sigma$. A bead is linked to each nearest neighbor in a triangular lattice by a finite extensible nonlinear elastic bond:

$$U_{b}=\frac{1}{2}k_{b}r_{\infty }^{2}\log\left[1-{\left(r/r_{\infty }\right)}^{2}\right]$$ (3)

where $k_{b}=30\varepsilon /\sigma^{2}$ and $r_{\infty }=1.5\sigma$. To model the stiffness propensity of the lipid molecule, a harmonic spring potential is applied between the head bead and the second tail bead:

$$U_{\text{bend}}=\frac{1}{2}k_{\text{bending}}{\left(r-4\sigma \right)}^{2}$$ (4)

where $k_{\text{bending}}=10\;\varepsilon /\sigma^{2}$. Under the control of these potentials, lipid molecules have been demonstrated to replicate experimentally observed behaviors such as the ability to self-assemble into a planar lipid bilayer or vesicle with a fluid-like phase under the temperature of $T=1.1\;\varepsilon /k_{B}$ [27,37], where $k_{B}$ is the Boltzmann constant. The bending stiffness κ of the lipid membrane is calculated based on the membrane fluctuation, κ=7.8ε. The membrane stretching modulus is 26.4 $\varepsilon /\sigma^{2}$. The potential for modeling interactions between the NP and lipid membrane can be expressed by the Lennard–Jones potential:

$$U_{\text{inter}}=4\varepsilon_{a}\left[{\left(\frac{b}{r}\right)}^{12}-{\left(\frac{b}{r}\right)}^{6}\right]$$ (5)

where $b=2^{\frac{7}{6}}\;\sigma$, $\varepsilon_{a}=0.2\;\varepsilon$, and the cutoff distance $r_{\mathrm{cut}}$ is equal to $3\times 2^{\frac{7}{6}}\;\sigma$. This interaction was chosen to simulate NPs with coatings that promote membrane wrapping.

### Simulation protocol

In our simulations, the unit mass is *m* and the mass for all beads is set to unity 5. The timestep ∆t was set as 0.005τ in the simulations, in which the time unit is defined as $\tau =\sqrt{\varepsilon /\left(m\sigma^{2}\right)}$. The temperature of the lipid bilayer was controlled as $1.1\;\varepsilon /k_{B}$ through a Langevin thermostat under which the membrane is in the fluid phase as found in living tissue. The time constant in the Langevin thermostat was set to be 0.5τ, 100 times of the timestep ∆t. Free membrane tension was applied through maintaining the in-plane pressure to be zero using a modified Berendsen method [31,34,38]. The time constant in the modified Berendsen barostat was set to be 5.0τ, 1,000 times of the timestep ∆t. Periodic boundary condition was applied in all 3 directions while the simulation box length in the out-of-plain direction was set to be sufficiently large, 400σ, to avoid unphysical self-interaction of the membrane during endocytosis of NPs. The physical interpretation of time and distance units in our simulations can be obtained through a comparison of computational and experimental results. First, the membrane thickness for mammalian cells is about 5 nm, while for our simulations, it is about 5σ, meaning that the distance unit σ can be 1 nm. Second, the diffusion coefficient of lipids in experiments is around 5 μm^2^/s while for this model it is 1×10^−2^ $\sigma^{2}/\tau$, leading to the physical interpretation of the time unit τ as 2 ns. The temperature of the membrane in the simulation is 1.1 $\varepsilon /k_{B}$, which is 310 K physically. Therefore, the energy unit ε is interpreted as $3.89\times 10^{-21}\;J$. In summary, the length unit σ is interpreted as 1nm, the time unit is interpreted as2ns, and the energy unit ε is interpreted as $3.89\times 10^{-21}\;J$. Based on the unit system above, the unit for stretching modulus of membrane $\varepsilon /\sigma^{2}$ is 5 mN/m. All the simulations have been performed via large-scale atomic/molecular massively parallel simulator (LAMMPS), an open-source package for molecular dynamics simulations.

### Cell culture

HepG-2 cells were cultured in DMEM containing 10% FBS and 1% antibiotics at 37 °C in a 5% CO_2_ atmosphere. In addition, HepG-2 cells (1 × 10^4^ per ml) were seeded in 6-well cell culture plates containing clean coverslips and incubated overnight in complete medium.

### In vitro cytotoxicity of MAuNS robots

HepG-2 cells with a density of 3,000 cells per well were seeded in a 96-well plate, followed by 12 h of incubation in 100 μl of DMEM with 10% FBS and 1% antibiotic. Then, various 100-μl fresh mediums containing MAuNSs with different concentrations of DOX (0 to 100 μg/ml) were added to the wells incubating HepG-2 cells after the initial medium was discarded. Subsequently, these samples were co-cultured with cells for 24 h. Next, after the second medium was discarded under magnetic field to avoid the magnetic particles from being removed, 100 μl of fresh medium containing 10 μl of CCK-8 was added to each well, followed by another 2 h of incubation. The absorbance was detected at 450 nm with a microplate reader. All the tests were repeated 8 times to reduce errors.

### Live/dead cell staining assay

HepG-2 cells were seeded onto a 96-well plate at a density of 5,000 cells per well and cultured with medium for 12 h. After cell attachment, 100 μl of medium with 20 μg of MAuNSs robots was replaced by the initial medium, followed by another 24 h of incubation. Then, the cells were washed 3 times with PBS and stained with Calcein-AM (5 μl) and PI (5 μl) solution in the PBS (5 ml) for 30 min at room temperature after the medium was removed. Finally, both cells were washed with PBS several times, and staining results were observed using an inverted fluorescence microscope at 494 and 535 nm.

### In vivo cancer therapy

Animal experiments were performed according to the guidelines of the Animal Care and Use Committee of the Tenth Affiliated Hospital of Southern Medical University. Liver tumor mice were divided into 4 groups when the tumor increased to 1.5 mm, as follows: one group of mice did not receive any treatment as a blank control group (*n* = 10), one group of mice received magnetron therapy; one group of tumor mice was treated with DOX, and one group of mice was treated with both magnetron therapy and DOX, and the intensity and frequency of the magnetic field were maintained at 200 mT and 16 Hz in all animal experiments.

## Data Availability

The data supporting the results of this study can be obtained from the corresponding authors upon reasonable request.

## References

[1] Wang L, Zhu X, Xu C, Jin D, Ma X. Artificial breakthrough of cell membrane barrier for transmembrane substance exchange: A review of recent progress. Adv Funct Mater. 2024;34(13):2311920.

[2] Xie J, Shen Q, Huang K, Zheng T, Cheng L, Zhang Z, Yu Y, Liao G, Wang X, Li C. Oriented assembly of cell-mimicking nanoparticles via a molecular affinity strategy for targeted drug delivery. ACS Nano. 2019;13(5):5268–5277. 31022341 10.1021/acsnano.8b09681

[3] Tang K, Tang Z, Niu M, Kuang Z, Xue W, Wang X, Liu X, Yu Y, Jeong S, Ma Y, et al. Allosteric targeted drug delivery for enhanced blood-brain barrier penetration via mimicking transmembrane domain interactions. Nat Commun. 2025;16(1):3410. 40210849 10.1038/s41467-025-58746-x PMC11986143

[4] Shi Z, Graber ZT, Baumgart T, Stone HA, Cohen AE. Cell membranes resist flow. Cell. 2018;175(7):1769–1779. 30392960 10.1016/j.cell.2018.09.054 PMC6541487

[5] Oroojalian F, Beygi M, Baradaran B, Mokhtarzadeh A, Shahbazi MA. Immune cell membrane-coated biomimetic nanoparticles for targeted cancer therapy. Small. 2021;17(12):2006484. 10.1002/smll.202006484 33577127

[6] Kakkar A, Traverso G, Farokhzad OC, Weissleder R, Langer R. Evolution of macromolecular complexity in drug delivery systems. Nat Rev Chem. 2017;1(8):0063. 10.1038/s41570-017-0063 PMC6613785 31286060

[7] Qiu C, Xia F, Zhang JZ, Shi Q, Meng Y, Wang C, Pang H, Gu L, Xu C, Guo Q, et al. Advanced strategies for overcoming endosomal/lysosomal barrier in nanodrug delivery. Research. 2023;6:0148. 37250954 10.34133/research.0148 PMC10208951

[8] Frey ML, Han S, Halim H, Kaltbeitzel A, Riedinger A, Landfester K, Lieberwirth I. Nanocarriers made of proteins: Intracellular visualization of a smart biodegradable drug delivery system. Small. 2022;18(15):2106094. 10.1002/smll.202106094 35224835

[9] Qin X, Yu C, Wei J, Li L, Zhang C, Wu Q, Liu J, Yao SQ, Huang W. Rational design of nanocarriers for intracellular protein delivery. Adv Mater. 2019;31(46):1902791. 10.1002/adma.201902791 31496027

[10] Liu Y, Zhang J, Tu Y, Zhu L. Potential-independent intracellular drug delivery and mitochondrial targeting. ACS Nano. 2021;16(1):1409–1420. 34920667 10.1021/acsnano.1c09456 PMC9623822

[11] Devine K, Villalobos E, Kyle CJ, Andrew R, Reynolds RM, Stimson RH, Nixon M, Walker BR. The ATP-binding cassette proteins ABCB1 and ABCC1 as modulators of glucocorticoid action. Nat Rev Endocrinol. 2023;19(2):112–124. 36221036 10.1038/s41574-022-00745-9

[12] van den Heuvel MGL, Dekker C. Motor proteins at work for nanotechnology. Science. 2007;317:333–336. 17641191 10.1126/science.1139570

[13] Si LY, Zhang SM, Guo HR, Luo W, Feng Y, du X, Mou F, Ma H, Guan J. Swarming magnetic Fe_3_O_4_@Polydopamine-tannic acid nanorobots: Integrating antibiotic-free superficial photothermal and deep chemical strategies for targeted bacterial elimination. Research. 2024;7:0438. 39086398 10.34133/research.0438 PMC11289052

[14] Ding Q, Huang S, Zhang Z, Yu D, Li M, He Q, Mei L. Integration of photodiagnosis and therapy guided by micro/nanorobots. Adv Mater. 2025;2420359. 10.1002/adma.202420359 40079099

[15] Li J, Esteban-Fernández de Ávila B, Gao W, Zhang L, Wang J. Micro/nanorobots for biomedicine: Delivery, surgery, sensing, and detoxification. Sci Robot. 2017;2(4):eaam6431. 31552379 10.1126/scirobotics.aam6431 PMC6759331

[16] Soto F, Wang J, Ahmed R, Demirci U. Medical micro/nanorobots in precision medicine. Adv Sci. 2020;7(21):2002203. 10.1002/advs.202002203 PMC7610261 33173743

[17] Liu K, Liu Q, Yang J, Xie C, Wang S, Tong F, Gao J, Liu L, Ye Y, Chen B, et al. Micromotor based mini-tablet for oral delivery of insulin. ACS Nano. 2022;17(1):300–311. 36546656 10.1021/acsnano.2c07953

[18] Li T, Liu Z, Hu J, Chen L, Chen T, Tang Q, Yu B, Zhao B, Mao C, Wan M. A universal chemotactic targeted delivery strategy for inflammatory diseases. Adv Mater. 2022;34(47):2206654. 10.1002/adma.202206654 36122571

[19] Jiang J, Hu J, Li M, Luo M, Dong B, Sitti M, Yan X. NIR-II fluorescent thermophoretic nanomotors for superficial tumor photothermal therapy. Adv Mater. 2025;37(10): Article 2417440. 39895191 10.1002/adma.202417440 PMC11899490

[20] Ma G, Liu Z, Zhu C, Chen H, Kwok RTK, Zhang P, Tang BZ, Cai L, Gong P. H_2_O_2_-responsive NIR-II AIE nanobomb for carbon monoxide boosting low-temperature Photothermal therapy. Angew Chem Int Ed Engl. 2022;61(36): Article e202207213. 35838004 10.1002/anie.202207213

[21] Smith SA, Selby LI, Johnston APR, Such GK. The endosomal escape of nanoparticles: Toward more efficient cellular delivery. Bioconjug Chem. 2018;30(2):263–272. 30452233 10.1021/acs.bioconjchem.8b00732

[22] Chen S, Sun X, Fu M, Liu X, Pang S, You Y, Liu X, Wang Y, Yan X, Ma X. Dual-source powered nanomotor with integrated functions for cancer photo-theranostics. Biomaterials. 2022;288: Article 121744. 35999081 10.1016/j.biomaterials.2022.121744

[23] Pan X, Xu D, Tang X, Liu N, You Y, Wang X, Yan X, Ma X, Chen X. Endocytosis-enabled construction of silica Nanochannels crossing living cell membrane for transmembrane drug transport. Adv Funct Mater. 2020;30(38):2002761.

[24] Sun M, Liu Q, Fan X, Wang Y, Chen W, Tian C, Sun L, Xie H. Autonomous biohybrid urchin-like microperforator for intracellular payload delivery. Small. 2020;16(23):1906701. 10.1002/smll.201906701 32378351

[25] Liu X, Yang S, Lyu X, Liu S, Wang Y, Li Y, Wang B, Chen W, Wang W, Guo J, et al. Instant preparation of ultraclean gold nanothorns under ambient conditions for SERS kit-enabled mobile diagnosis. Anal Chem. 2021;93(49):16628–16637. 34855357 10.1021/acs.analchem.1c04099

[26] Wang B, Kostarelos K, Nelson BJ, Zhang L. Trends in micro-/nanorobotics: Materials development, actuation, localization, and system integration for biomedical applications. Adv Mater. 2020;33(4):2002047. 10.1002/adma.202002047 33617105

[27] Cooke IR, Deserno M. Solvent-free model for self-assembling fluid bilayer membranes: Stabilization of the fluid phase based on broad attractive tail potentials. J Chem Phys. 2005;123(22): Article 224710. 16375498 10.1063/1.2135785

[28] Chen L, Xiao S, Zhu H, Wang L, Liang H. Shape-dependent internalization kinetics of nanoparticles by membranes. Soft Matter. 2016;12(9):2632–2641. 26853682 10.1039/c5sm01869b

[29] Reynwar BJ, Illya G, Harmandaris VA, Müller MM, Kremer K, Deserno M. Aggregation and vesiculation of membrane proteins by curvature-mediated interactions. Nature. 2007;447:461–464. 17522680 10.1038/nature05840

[30] Ruiz-Herrero T, Velasco E, Hagan MF. Mechanisms of budding of nanoscale particles through lipid bilayers. J Phys Chem B. 2012;116(32):9595–9603. 22803595 10.1021/jp301601g PMC3428956

[31] Shen Z, Ye H, Li Y. Understanding receptor-mediated endocytosis of elastic nanoparticles through coarse grained molecular dynamic simulation. Phys Chem Phys. 2018;20(24):16372–16385. 10.1039/c7cp08644j 29445792

[32] Shen Z, Ye H, Yi X, Li Y. Membrane wrapping efficiency of elastic nanoparticles during endocytosis: Size and shape matter. ACS Nano. 2019;13(1):215–228. 30557506 10.1021/acsnano.8b05340

[33] Shi X, von dem Bussche A, Hurt RH, Kane AB, Gao H. Cell entry of one-dimensional nanomaterials occurs by tip recognition and rotation. Nat Nanotechnol. 2011;6:714. 21926979 10.1038/nnano.2011.151 PMC3215144

[34] Sun J, Zhang L, Wang J, Feng Q, Liu D, Yin Q, Xu D, Wei Y, Ding B, Shi X, et al. Tunable rigidity of (polymeric core)-(lipid shell) nanoparticles for regulated cellular uptake. Adv Mater. 2015;27(8):1402–1407. 25529120 10.1002/adma.201404788

[35] Vácha R, Martinez-Veracoechea FJ, Frenkel D. Receptor-mediated endocytosis of nanoparticles of various shapes. Nano Lett. 2011;11(12):5391–5395. 22047641 10.1021/nl2030213

[36] Xiong K, Zhao J, Yang D, Cheng Q, Wang J, Ji H. Cooperative wrapping of nanoparticles of various sizes and shapes by lipid membranes. Soft Matter. 2017;13(26):4644–4652. 28650048 10.1039/c7sm00345e

[37] Li Y, Stroberg W, Lee T-R, Kim HS, Man H, Ho D, Decuzzi P, Liu WK. Multiscale modeling and uncertainty quantification in nanoparticle-mediated drug/gene delivery. Comput Mech. 2014;53(3):511–537.

[38] Guan Z, Wang L, Lin J. Interaction pathways between plasma membrane and block copolymer micelles. Biomacromolecules. 2017;18(3):797–807. 28125207 10.1021/acs.biomac.6b01674

