# Supplementary material for: Mechanical Agitation-Assisted Transmembrane Drug Delivery by Magnetically Powered Spiky Nanorobots
Source: Research (Wash D C). 2025 Aug 13;8:0768. doi: 10.34133/research.0768 (PMC12349924; doi:10.34133/research.0768)
Supplement: Supplementary 1 — Figs. S1 to S16 Movies S1 to S7 [file research.0768.f1.zip › Supplementary Materials_Rev1.docx]

SUPPLEMENTARY MATERIALS

Title

Mechanical Agitation Assisted Transmembrane Drug Delivery by Magnetically Powered Spiky Nanorobots

**Authors**

Xiaojia Liu^1,2†^, Zihan Xu^2†^, Yanan Che^3^, Zichang Guo^2^, Dongdong Jin^2^, Qianqian Wang^4,5^, Ning Liu^6*^, Xing Ma^2*^, Zhilu Yang^1*^

**Affiliations**

^1^ Dongguan Key Laboratory of Smart Biomaterials and Regenerative Medicine, The Tenth Affiliated Hospital, Southern Medical University, Dongguan, Guangdong, 523059, China

^2^ Sauvage Laboratory for Smart Materials, School of Integrated Circuits, Harbin Institute of Technology (Shenzhen), Shenzhen, 518055, China

^3^ Department of Mechanical and Automation Engineering, The Chinese University of Hong Kong, Hong Kong, SAR 999077, China

^4^ Jiangsu Key Laboratory for Design and Manufacturing of Precision Medicine Equipment, School of Mechanical Engineering, Southeast University, Nanjing, 211189, China

^5^ School of Mechanical Engineering, Southeast University (Jiulonghu Campus), Jiangning District, Nanjing, 211189, China.

^6^ School of Aerospace Engineering and Applied Mechanics, Tongji University, Shanghai, 200092, China

^†^These authors contributed equally to this work.

^*^Address correspondence to: 21019@tongji.edu.cn (N.L); [maxing@hit.edu.cn (X.M);](mailto:maxing@hit.edu.cn;) zhiluyang1029@smu.edu.cn (Z.Y)


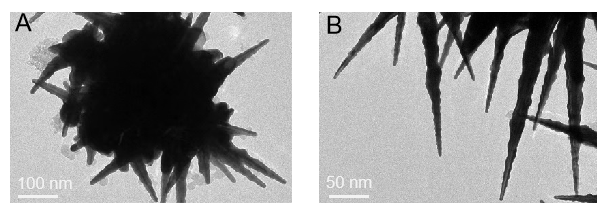


**Fig. S1.** TEM image of (A) an AuNS and (B) magnification of its spiky structure.


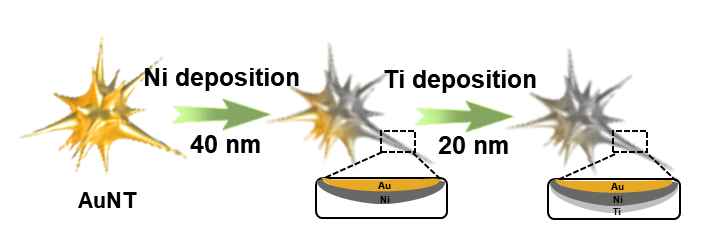


**Fig. S2.** Schematic illustration of physical deposition of Ni and Ti nanolayers on the AuNS to yield MAuNSs robots.


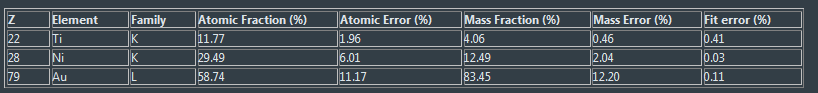


**Fig. S3.** Elemental analysis of MAuNSs by EDS.


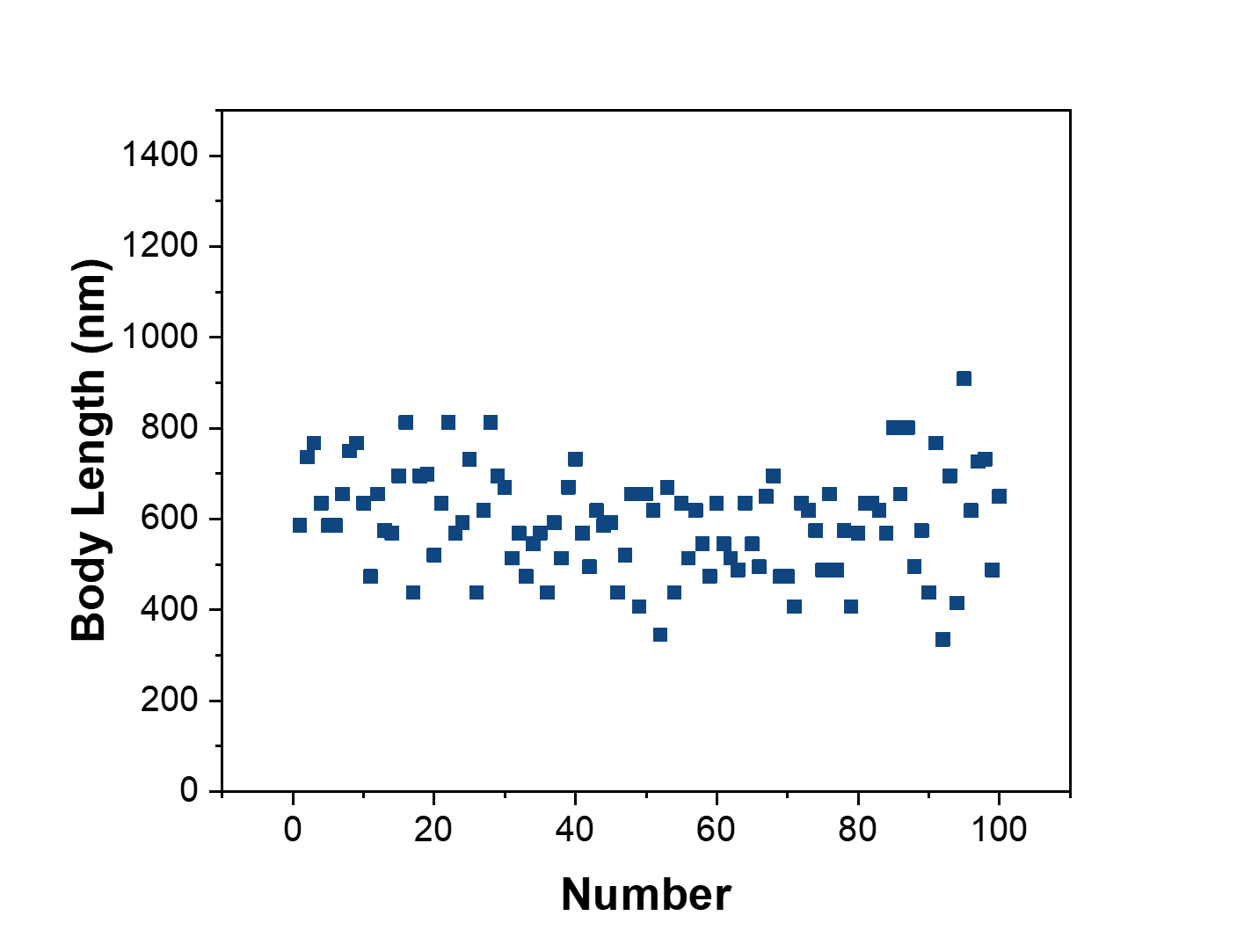


**Fig. S4.** Statistics of the body length of the MAuNSs (n=100).


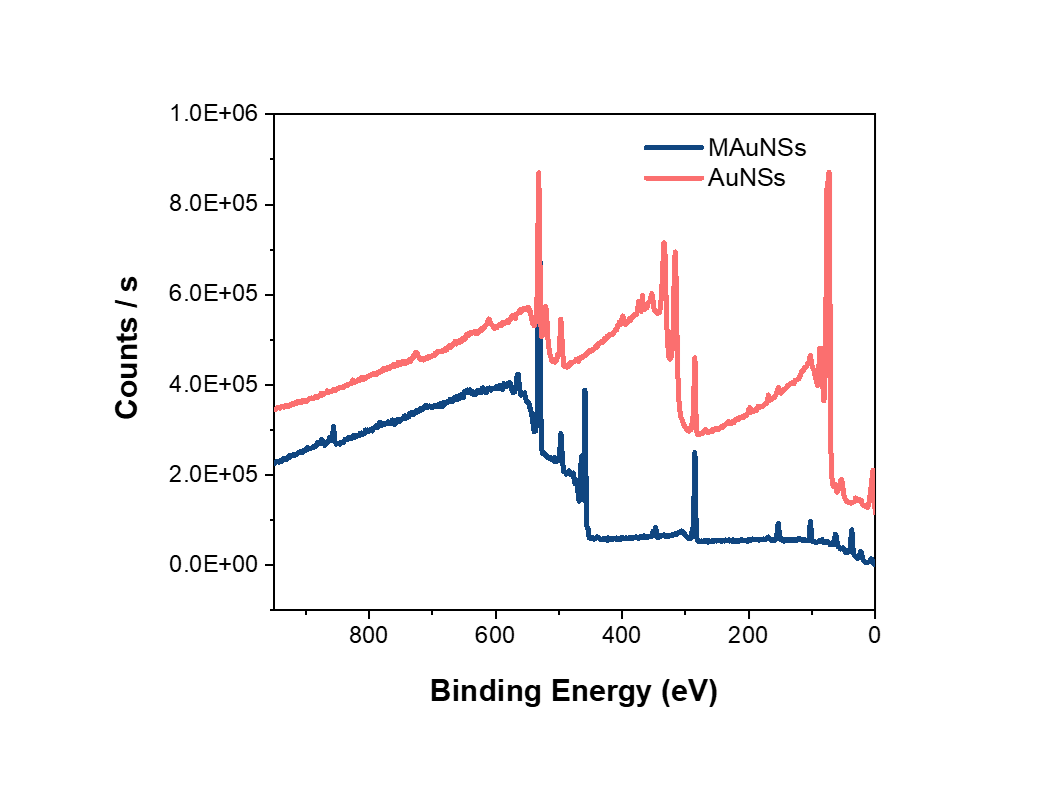


**Fig. S5.** XPS spectra of AuNSs and MAuNSs


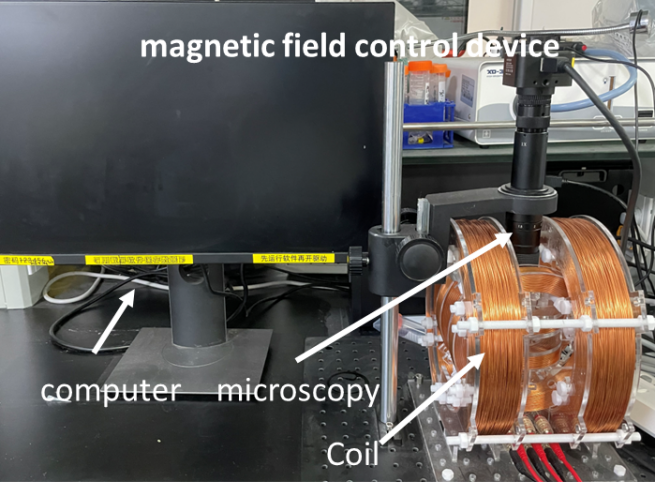


**Fig. S6.** Three-dimensional device: Pictures of three-dimensional magnetic field control devices composed of Helmholtz coil and digital microscopy.


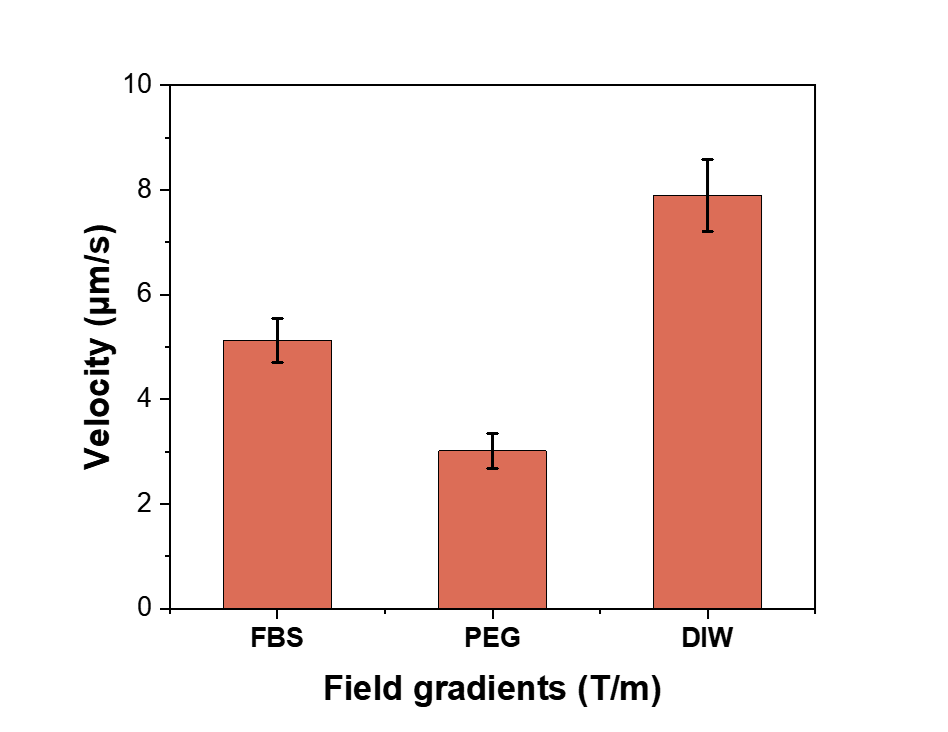


**Fig. S7.** Velocity of the MAuNSs within different liquid environments (FBS、PEG and deionized water). (Error bars indicate standard deviation, n= 50)

*
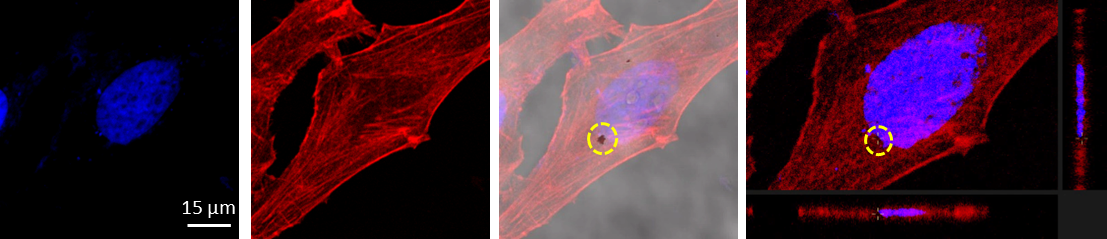
*

**Fig. S8.** Confocal laser scanning microscopy (CLSM) images of cells incubated with MAuNSs robots.


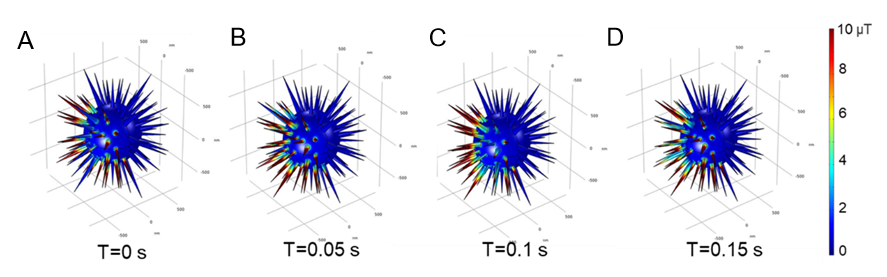


**Fig. S9.** Simulation of magnetic flux density of a MAuNSs robot at different times under a rotating magnetic field at different time: (A) T=0 s, (B) T=0.05 s, (C) T=0.1 s, (D) T=0.15 s.


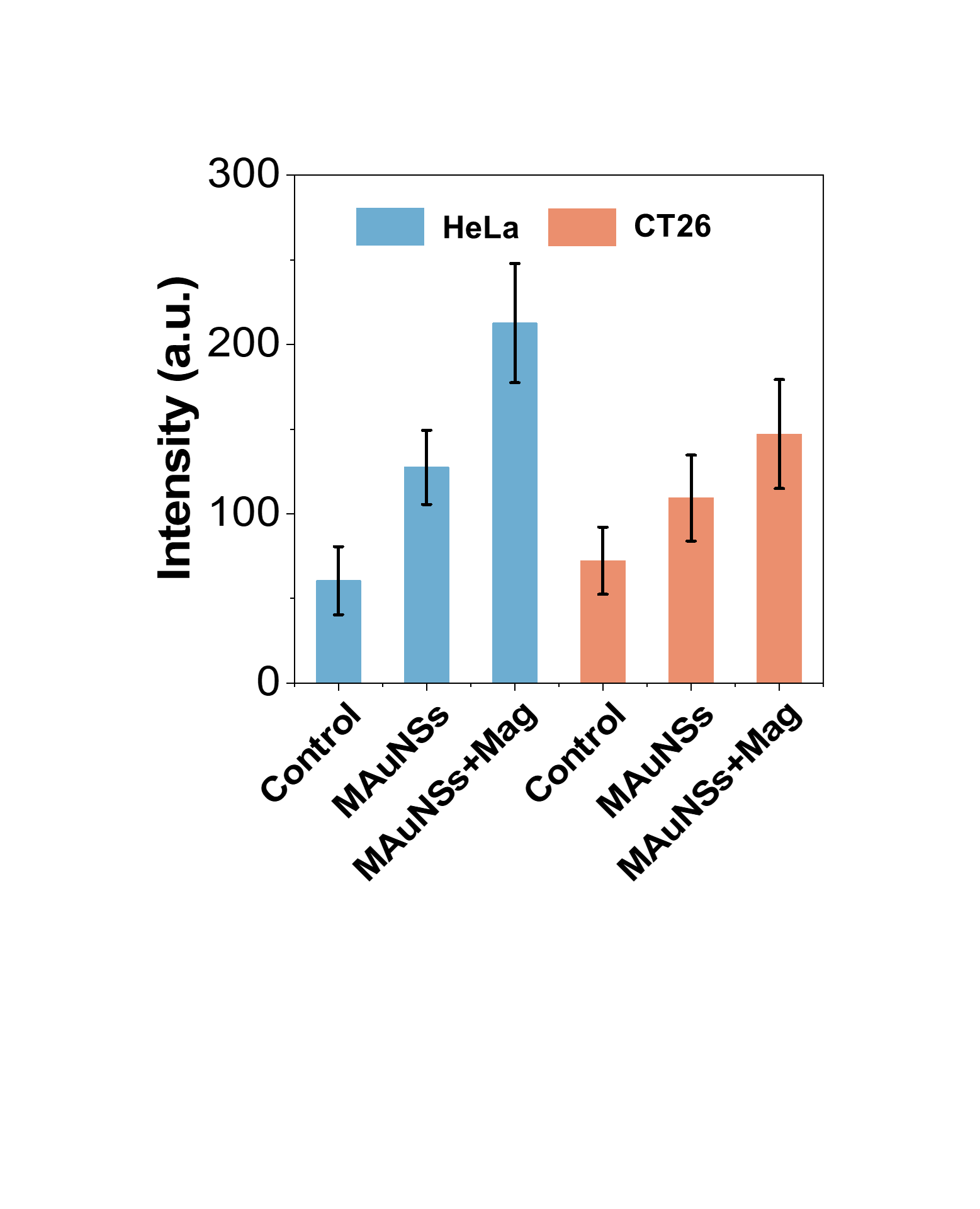


**Fig. S10.** Fluorescence intensity analysis of drug molecules entering tumor cells (HeLa and CT26) under different conditions.

**
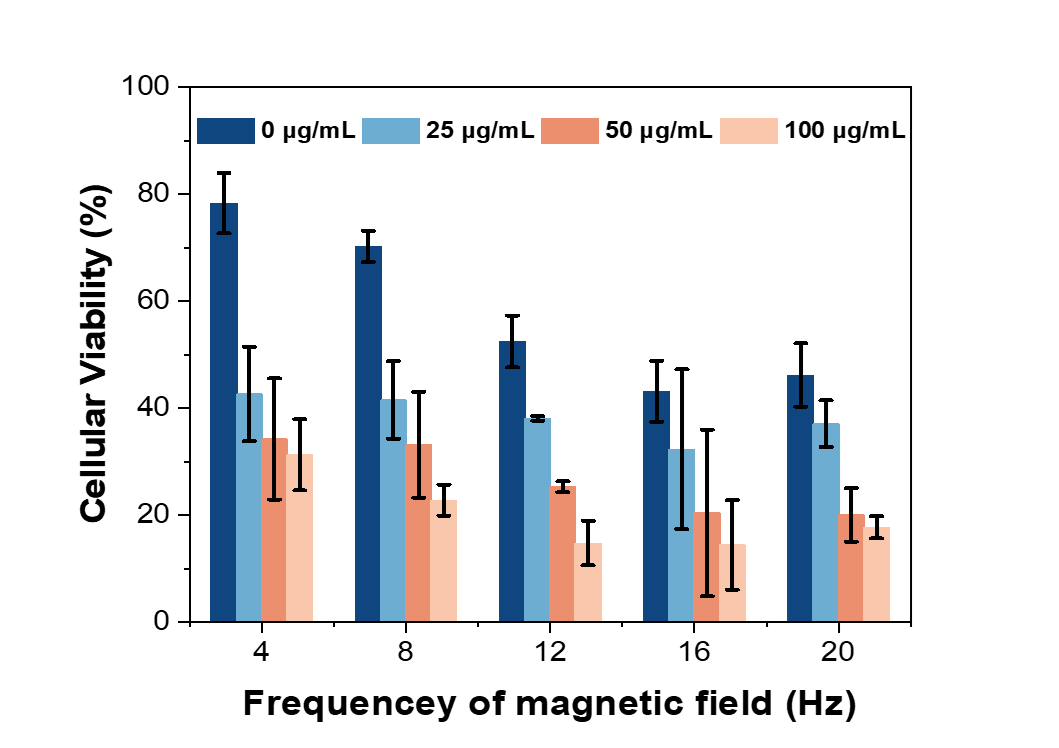
**

**Fig. S11.** CCK-8 test showing cellular viability of HepG-2 cells incubated with MAuNSs robots, and then treated with magnetic field of different frequencies. with additiona of different concentration (0, 25, 50, 100 μg/mL) of DOX. (Error bars indicate standard deviation, n= 8)


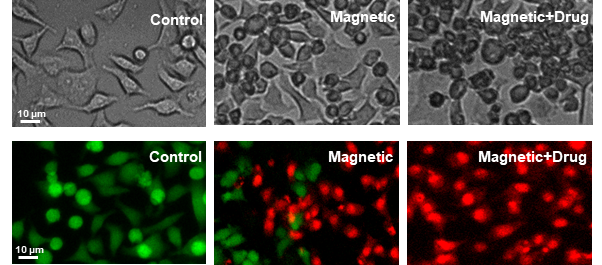


**Fig. S12.** Live/Dead cell staining with different treatments (Control, Magnetic and Magnetic+ Drug; Frequency of magnetic field:12 Hz; DOX concentration: 25 μg/mL).


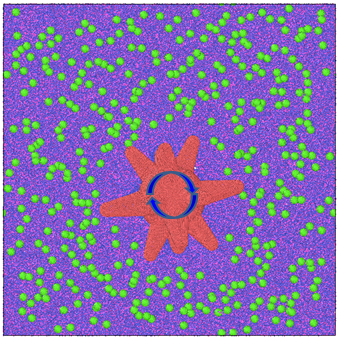


**Fig. S13.** Schematic showing small cargo (green dots) and nanospike robots (red) models used in the simulation.


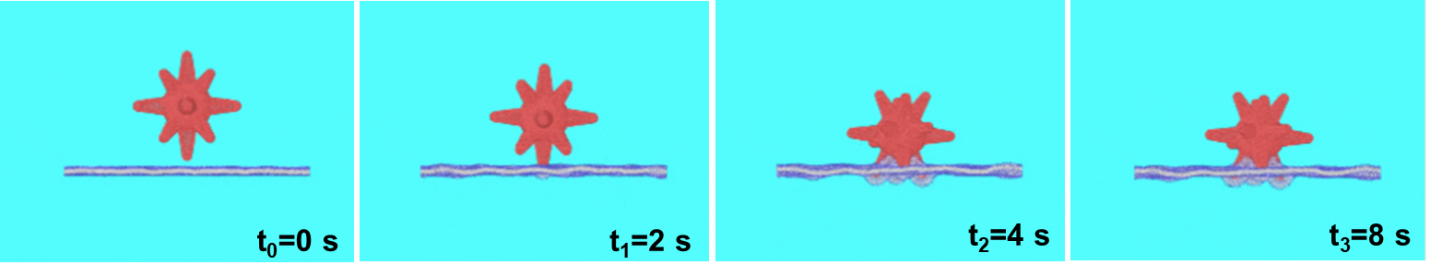


**Fig. S14.** Screenshots of the dynamic behavior of a MAuNSs robot interaction with the cell membrane at different times (t_0_=0 s, t_1_=2 s, t_2_=4 s, t_3_=8 s). (Torque =0)


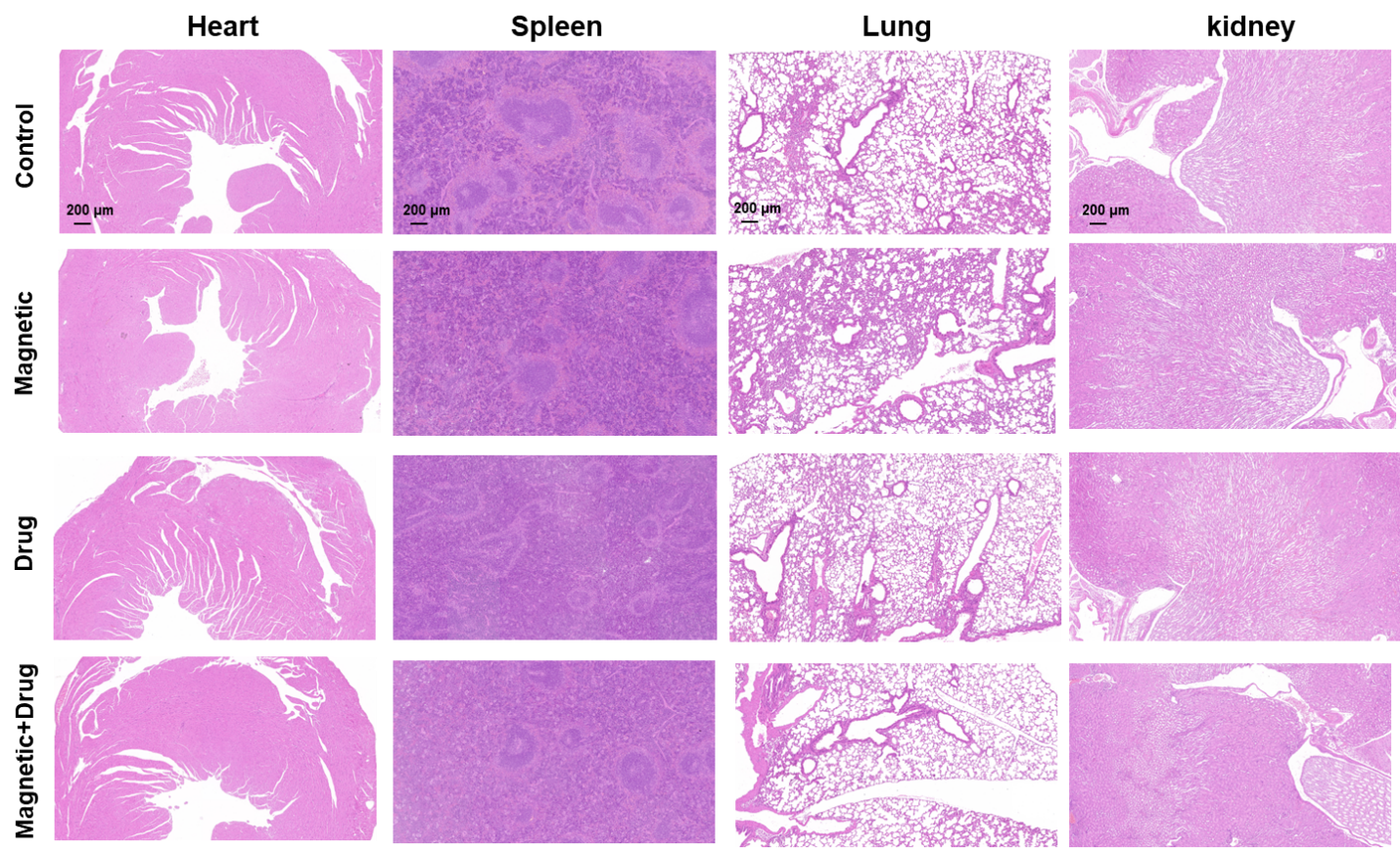


**Fig. S15.** In vivo safety evaluation. H&E staining of the heart, spleen, lung and kidney tissue slices from tumor mice after treating for 21 days.


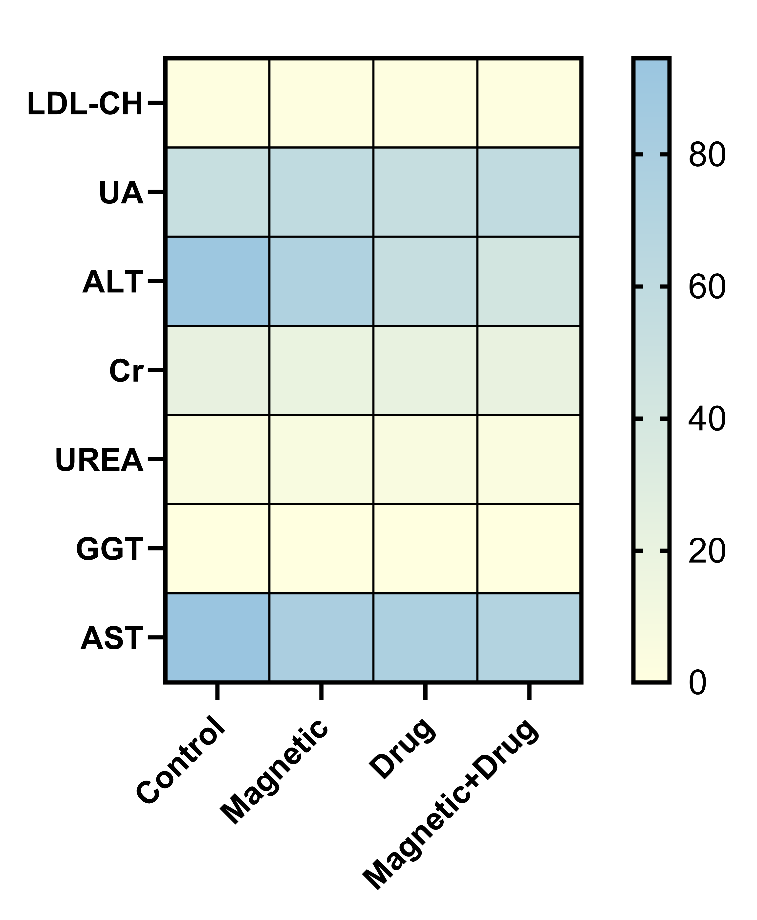


**Fig. S16.** In vivo blood safety evaluation. Blood biochemical data from tumor mice with

different treatment conditions.
